# The Anatomy of the Human Eye

**Published:** 1834-07-01

**Authors:** 


*The Anatomy of the Human Eye.* By JOHN DALRYMPLE, Assistant Surgeon to the London Ophthalmic Infirmary.—London, 1834. 8vo. pp. 294; Plates.

A GOOD monograph on the structure of the eye has for some years been a desideratum in our medical literature: the investigation of this organ constitutes a separate department of anatomy, and cannot be satisfactorily conducted within the limits of an ordinary anatomical system, or an introduction to a pathological treatise. The principal materials for such a work must necessarily be derived from the labours of preceding authors, but the task of collecting and weighing their statements and opinions is, if judiciously performed, one of considerable difficulty and great utility: the subject also still affords sufficient scope for original observation in some of its minuter details. Mr. Dalrymple has shewn great industry and judgment in the collection of his materials, and has taken nothing upon trust, but has been careful to verify or correct the statements of other anatomists by his own observation: he has also introduced several interesting descriptions and ingenious opinions of his own. We give one or two extracts merely as specimens of the work. The following is our author's account of the foramen of Soemmering.

"In the description of the sclerotic, I had occasion to notice the lamina cribrosa, and the point where the optic nerve enters, as not being placed in the direct centre of the eye; or, in other words, not in the exact axis of vision, but being rather to the inner or nasal side of the organ. If the globe of a recent human eye be divided by a transverse section, and, whilst immersed in water, the vitreous body along with the anterior section be removed, the posterior portion of the retina will be brought into full view; which, notwithstanding the delicacy of its texture, will remain undisturbed, and fairly exhibit the lateral entrance of the nerve. From this point we at once trace a small elliptical fold, that extends transversely to the very axis of vision; where is observed in its centre a small dark spot, not perfectly round, but rather oval, like the fold in which it is placed; it is also slightly depressed, and surrounded by a yellow circumference. This spot is what Soëmmering discovered, and described as the 'foramen et limbum luteum,' and which, during several years, was considered peculiar to man: but Cuvier has since remarked it to prevail in some of the genus, 'quadrumana;' and by Dr. Knox it has subsequently been observed in the eyes of certain lizards, and in the chamelion; the last mentioned author has denied the appearance of a fold in the human eye, and treats its existence only as a post-mortem result. I however believe the fold to be essential to the formation of the dark-coloured spot described by Soëmmering.

"A contrariety of opinions has existed in reference to this point. The illustrious author of the discovery, by the very name he has applied to it, considers it as a real aperture, surrounded by a yellowish coloured circumference; while Blumenbach attributes to it an expansive and contractile power, for the purpose of regulating, under certain circumstances, the admission of rays of light. Sir C. Bell denies the existence of a real foramen, the apparent aperture being produced by the transparency of the retina at this point, while its remaining portions become uniformly opaque. Blainville, in his Principes d'Anatomie Comparée, though generally correct in his description of this appearance, falls, I believe, into a similar error: 'Un petit enfoncement plus ou moins ovalaire, translucide au milieu, autour duquel se plisse un peu la rétine, qui l'on remarque dans cette membrane, en quelque distance en dehors de l'entrée du nerf optique, dans l'axe meme du globe de l'œil.' For myself, I am disposed to think that the existence of the apparent aperture in question may be explained on other grounds than that of the transparency of the central spot. I have remarked that it is not, as Soëmmering has represented it, a perfectly circular point, but that its figure is oval, corresponding in this respect with the plica, in the centre of which it is placed: this is also stated by Blainville. This fold is formed by a duplicature of the retina, or rather by the union of two minute plicæ, united at their respective extremities, and leaving a depression between them in their centre. Their extremities thus joined extend on the one side to the entrance of the optic nerve, while on the other, or temporal side, they are lost by expanding into the general surface of the retina: the extreme length of these folds is about one and a half lines, in breadth nearly one line. This appearance I have repeatedly observed in the human eye when immersed in water, as I have already described. If the subject under observation be carefully frozen, and neatly separated by a vertical section through the centre of the folds, it will be still more distinctly shewn, and exhibit the central depression, consisting of a single layer of retina only; whilst the folds, consisting of two portions of membrane doubly opaque, and standing out in relief, throw it into shade, the dark pigment behind, adding a still greater depth of colour, increases the effect of the shadow.

"This conclusion is still further sustained by the appearances exhibited by a preparation in my possession, in which, by long immersion in dilute spirit of wine, the central depression has partially unfolded, shewing the continuity of the retina between the folds as perfectly opaque as in any other portion of its surface. The yellow circumference, or 'limbum luteum,' has been also blanched by the spirit, a result, as to colour, equally produced on the grey matter of the brain by long continued immersion.

"The opinions which I have here ventured to submit to the profession are the result of dissections made six years ago, the vouchers for which I still retain in the preparations then made:

they have been confirmed by recent and continued examination of this point; and, as the inspections afforded me, on these occasions, were made under circumstances the most favorable, as regarded the recent state of the organ, I am led to speak with increased confidence of the nature of the appearances described in the preceding paragraphs." (P. 75.)

Mr. Dalrymple makes some remarks on the discovery of Dr. Jacob, who, it appears, does not understand the mystery of his own membrane: Is it wonderful, then, that others should have been slow in apprehending it? nay, that some heretically-disposed persons should have started doubts whether it were anything or nothing?

"Dr. Jacob," observes our author, "does not assert the identity of this membrane with serous tissues in general, although some confusion may arise from his likening it to the lining membrane of serous cavities. It is also evident, that the discoverer believed it to be a single membrane. From observations made on the human eye, in connexion with other experiments on the eyes of animals, I am induced to consider it as *a double reflected serous membrane*." (P. 95.)

The following, according to Mr. Dalrymple, is the true anatomy of the membrane in question.

"At the point of entry of the optic nerve this delicate tissue first commences; it proceeds concentric with, lines, and is attached to the internal surface of the choroid, as far as the ciliary circle, and 'ora serrata;' thence doubling on itself, it is reflected upon the outer surface of the retina, to which it is loosely adherent, forming that exquisite structure first observed and described by Dr. Jacob: in this way it proceeds back to the optic nerve, where it is again continuous with the portion lining the choroid. The latter portion, or the choroidal reflection, wheresoever it comes in contact with the pigment, is stained by it, though it does not allow the colouring matter to transude. Thus, instead of its being a single layer, I think we are enabled to establish, that it is a double and reflected tissue, forming a closed sac, and presenting the peculiarities, anatomical and physiological, common to all such membranes as line serous cavities.

"Certain morbid conditions of the eye would seem to point out the existence of a serous membrane at this part, and of these the most remarkable is a peculiar form of 'gutta serena,' mentioned by Mr. Ware and others, who, in several instances after death, have observed a fluid effused between the choroid and retina. The former gentleman, by puncturing the sclerotic and choroid in some cases, gave immediate relief by removing the pressure on the retina produced by this morbid accumulation. In some rare instances the retina has been found ossified; and Meckel is of opinion, that Jacob's membrane is the seat of this affection. Ossification of serous textures does not unfrequently occur in other parts of the body; as, for instance in the pleura costalis, in the pericardium, on the peritoneal surface of the spleen, &c. It is also sometimes observed on the arachnoid surface of the dura mater. I know of no well authenticated instance, in which the medullary matter of the brain has been converted into bone, although I believe a rare case of ossified brain has been somewhere recorded.

"A disease which in its early stage bears some resemblance to fungus in the eye, presents a confirmation of the existence of this serous tissue: hitherto these cases have been described as effusions of lymph from the internal surface of the choroid. Is it not more probable that such effusions have arisen by secretion from a serous surface?" (P. 99.)

This work is a valuable contribution to anatomical literature, and we strongly recommend it to the student of ophthalmic surgery.

